# Seasonal Theanine Accumulation and Related Gene Expression in the Roots and Leaf Buds of Tea Plants (*Camellia Sinensis* L.)

**DOI:** 10.3389/fpls.2019.01397

**Published:** 2019-10-30

**Authors:** Fang Li, Chunxia Dong, Tianyuan Yang, Jingzhen Ma, Shupei Zhang, Chaoling Wei, Xiaochun Wan, Zhaoliang Zhang

**Affiliations:** State Key Laboratory of Tea Plant Biology and Utilization, Anhui Agricultural University, Hefei, China

**Keywords:** *Camellia sinensis*, theanine, biosynthesis and transport, seasonal regulation, theanine transporter, CsLHT

## Abstract

Theanine, a unique and abundant non-proteinogenic amino acid in tea, confers to the tea infusion its umami taste and multiple health benefits. Its content in new tea shoots is dynamic in winter and spring. However, its seasonal accumulation pattern and the underlying regulation mechanism of tea plants remain largely unknown. In this study, we measured the theanine contents in the roots and leaf buds of 13 tea cultivars at four time points from winter to spring (Dec. 12, Mar. 1, Mar. 23, and Apr. 13). We found theanine accumulated significantly in the roots to as high as ∼6% dry weight. We found theanine content in the roots was constant or slightly decreased on Mar. 1 compared with Dec.12 but increased consistently on Mar. 23 and then decreased on Apr. 13 in all 13 cultivars. In the leaf buds, theanine content kept increasing from Mar. 1 to Mar. 23 and decreasing from Apr. 13 in most of the 13 cultivars, meaning it was probably both season- and developmental stage-dependent. The expression of theanine biosynthesis and amino acid transporter genes in the roots and buds at the four time points was then examined. The correlation analyses between the gene expression and theanine content suggested the expression of theanine-biosynthesis genes was generally and negatively correlated with theanine content; however, the expression of amino acid transporter genes including *CsLHT* was generally and positively correlated with theanine contents. Finally, we showed that *CsLHT* has theanine transport activity. Taken together, this study provided insight into the seasonal regulation of theanine biosynthesis and transport in tea plants during winter and spring.

## Introduction

Tea plants are unique; an abundance of health-promoting and pleasant flavor-conferring components, including catechins, caffeine, aroma, and theanine, accumulate in a single tea leaf. This makes tea one of the most popular beverages in the world. Theanine, a non-proteinogenic amino acid, is the most abundant free amino acid in tea, and it confers the tea infusion its umami taste (Ashihara, 2015). Recently, it was shown that theanine derivatives are also critical components of the aroma of oolong tea and large-leaf yellow tea (Guo et al., 2018; Guo et al., 2019). Impressively, many health-promoting effects of theanine, such as its anti-anxiety, anti-tumor, neuron-protecting, and memory improving properties alongside its antagonistic effect on the negative action of caffeine among other things, have been studied intensively (Sharma et al., 2018). Therefore, theanine content in tea leaves is highly related to the quality and price of teas, especially green teas (Yamaguchi and Ninomiya, 2000).

The substrates for theanine biosynthesis are glutamate (Glu) and ethylamine (EA). The enzyme catalyzing theanine synthesis is theanine synthetase (TS) (Sasaoka et al., 1965). Recently Wei and his colleagues identified the *TS* gene (*CsTSI*) in the tea plant (*Camellia sinensis* L.) (Wei et al., 2018). However, Cheng et al. showed that the glutamine synthetases (GS) of tea plants and *Arabidopsis* can also catalyze theanine biosynthesis from Glu and EA (Cheng et al., 2017). In bacteria, theanine can be synthesized from glutamine (Gln) and EA by gamma-glutamyl*transpeptidase* (GGT) (Suzuki and Kumagai, 2002; Suzuki et al., 2002; Yao et al., 2006; Shuai et al., 2010).

**Figure 1:**
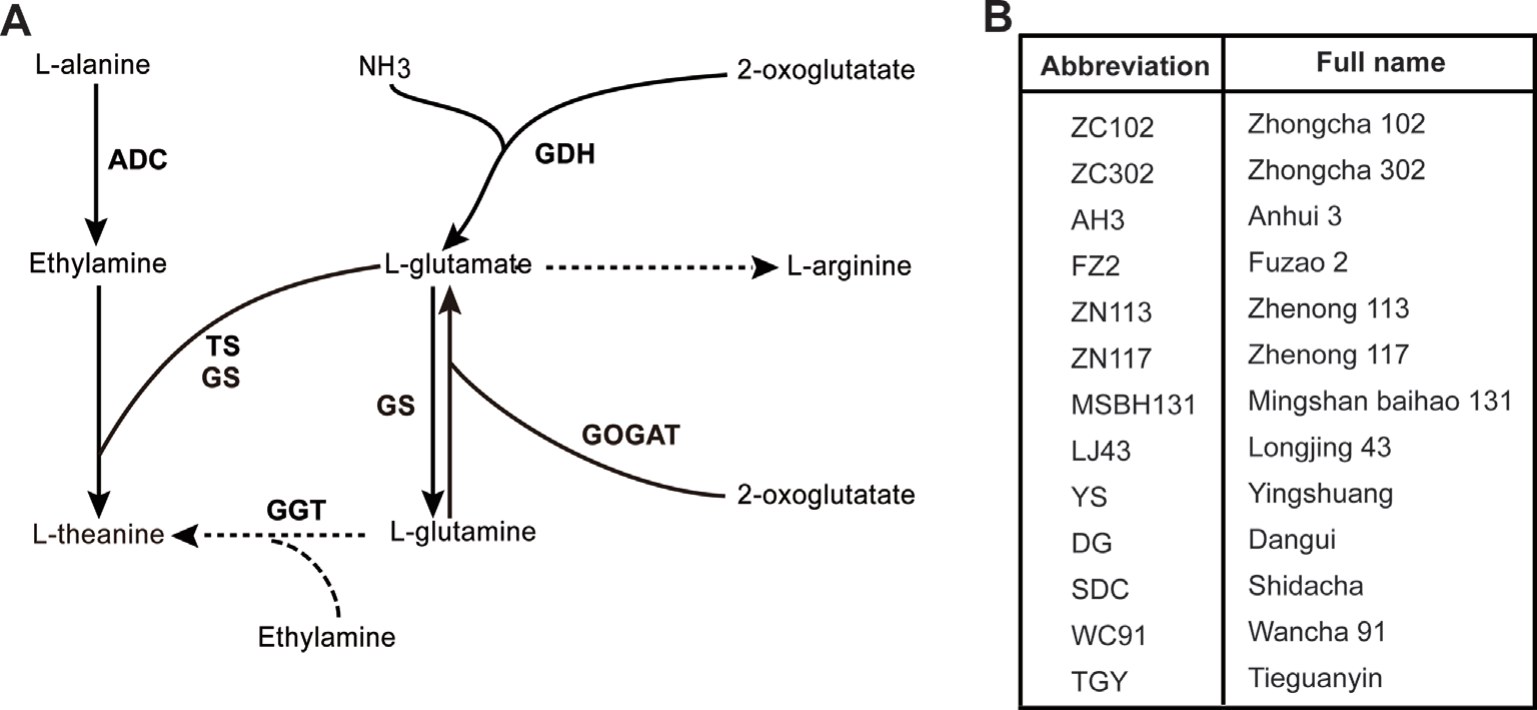
Putative theanine biosynthesis pathway in tea plant and cultivars used in this study. **(A)** Putative theanine biosynthesis pathway and the enzymes in tea plant. GDH, glutamate dehydrogenase; GOGAT, glutamate synthase; TS, theanine synthetase; GS, glutamine synthetase; GGT, glutamyltranspeptidase; *ADC*, alanine decarboxylase. **(B)** The 13 tea cultivars used in this study. All cultivars were 8-year-olds and were grown in the same tea plantation under the same management.

EA is derived from the decarboxylation of alanine catalyzed by alanine decarboxylase (AIDA) (Takeo, 1978). Arginine decarboxylase (ADC) was suggested to act as AIDA in tea plants (Shi et al., 2011). Glu is synthesized from Gln and ɑ-ketoglutarate under the catalysis of glutamate synthase (GOGAT). Glu can also be synthesized from ammonium and ɑ-ketoglutarate by Glu dehydrogenase (GDH) in tea plants (Takeo, 1979). The biosynthesis pathway and the enzymes involved in the process are illustrated in **Figure 1A**. Genes coding for enzymes in theanine biosynthesis have been widely identified in tea plants along with the completion of the genome sequencing (Xia et al., 2017; Wei et al., 2018).

Theanine catabolism is catalyzed by theanine hydrolase in tea plants (Tsushida and Takeo, 1985). This process occurs mainly in leaves and is regulated by light (Kito et al., 1968). However, the gene encoding theanine hydrolase has not been identified yet.

In general, plants assimilate nitrogen (N) into Glu, Gln, Asp, and Asn as the main transport forms of N (Fisher et al., 1998). Tea plants are unique for the assimilation and storing of most N in the form of theanine in their roots (Oh et al., 2008). Theanine can account for 60–70% of free amino acid content in the roots and new shoots of tea plants (Takeo, 1981). Tea plants mainly transport Gln, Theanine, and Glu from root to shoot (Oh et al., 2008). Thus, theanine is also a transport in the form of N in tea plants. However, transporters that mediate theanine transport have not been identified in tea plants. As theanine has a similar structure to Gln, amino acid transporters transporting Gln likely have theanine transport activity.

In plants, there are two superfamilies of amino acid transporters: the amino acid/auxin permease (AAAP) and the Amino acid-Polyamine-Choline transporter (APC) family (Tegeder et al., 2007). The AAAP superfamily consists of amino acid permeases (AAPs), lysine, and histidine transporters (LHTs), proline transporters (ProTs), ɣ-aminobutyric acid transporters (GATs), auxin transporters (AUXs), and aromatic and neutral amino acid transporters (ANTs). Vesicular aminergic-associated transporters (VAATs) were recently identified and were shown to modulate amine neurotransmission in animals (Larhammar et al., 2014). The homologs of animal VAATs were identified in the potato genome (Ma et al., 2016). Amino acid transporter-like (ATLs) were also identified in potato and soybean (Cheng et al., 2016; Ma et al., 2016). Both VAATAs and ATLs were classified into the AAAP superfamily. The APC superfamily includes cationic amino acid transporters (CATs), amino acid/choline transporters (ACTs), and polyamine H^+^-symporters (PHSs) (Tegeder et al., 2007). Recently, it is reported that members of the Usually Multiple Acids Move In and out Transporters family (UMAMIT) can also transport amino acids (Muller et al., 2015; Besnard et al., 2016, 2018). Members of AAPs, LHTs, ANTs, CATs, and UMAMITs are reported to have varied affinities for Gln. The conserved homologs of these amino acid transporters are the candidate transporters mediating theanine transport from root to new shoot as theanine has a similar structure to Gln.

Previous studies have suggested that theanine is primarily synthesized in the root in the winter (Konishi and Kasai, 1968; Konishi and Takahashi, 1969). Ruan et al. (2012) also observed that theanine concentration in xylem sap increased in the spring. However, how the biosynthesis and transport are regulated during winter and spring is largely unknown. In this study, we investigated the dynamic changes of theanine content and expression of related genes were in the roots and buds of 13 cultivars at four time points of tea leaf bud development. The results demonstrated theanine accumulation patterns in the roots and buds during winter and spring and also provided insight into the regulation of theanine biosynthesis and transport.

## Materials and Methods

### Plant Samples

Leaf buds and unlignified lateral roots were collected from 13 tea plant (*Camellia sinensis* L.) cultivars grown in the Guohe Tea Plantation (Lujiang, Anhui, China) on December 12, 2017 (Dec. 12), March 1, 2018 (Mar. 1), March 23, 2018 (Mar. 23), and April 13, 2018 (Apr. 13). Leaf buds of the representative shoots within the top crown area were sampled. These representative shoots were at a similar developmental stage as the same cultivar. These cultivars included Zhongcha 102 (ZC102), Zhongcha 302 (ZC302), Anhui 3 (AH3), Fuzao 2 (FZ2), Zhenong 113 (ZN113), Zhenong 117 (ZN117), Mingshanbaihao 131 (MSBH131), Longjing 43 (LJ43), Yingshuang (YS), Dangui (DG), Shidacha (SDC), Wancha 91 (WC91), and Tieguanyin (TGY). These tea plant cultivars were all 8-year-old stem cutting clones and the growth status was similar. These tea plants were routinely managed: they were pruned for ∼50 cm and given 450 kg of inorganic compound fertilizer (N: P_2_O_5_: K_2_O, 15:15:15) and 150 kg urea per hectare in May, 2017; given 2,250 kg soybean cake fertilizer and 450 kg urea per hectare in October, 2017. The collected samples were immediately frozen in liquid nitrogen and stored at -80°C.

### Determination of L-Theanine Content

Theanine was exacted and detected as previously described (Tai et al., 2015) with slight modification. The samples were grounded into a fine powder in liquid nitrogen before being freeze-dried. Then, 50 mg root or 200 mg bud was used to extract theanine, using 5 ml double distilled water, by boiling at 100°C for 20 min and cooling to room temperature. After centrifuging at 6,000 rpm for 10 min, the supernatant was filtered through a 0.22-µm membrane and analyzed by a Waters e2695 HPLC system equipped with 2489 UV/Vis detector. A reverse-phase C18 column (5 µm, 250 mm × 4.6 mm) was used and the column temperature was set at 28°C. The wavelength was set at 210 nm. The mobile phase A was water and phase B was 100% acetonitrile. The gradient elution was as follows: 0 min, 100% A, 0% B; 7 min, 100% A, 0% B; 9 min, 40% A, 60% B; 15 min, 100% A, 0% B; 20 min, 100% A, 0% B. The HPLC system was injected with 10 µl of the extraction for measurement. The amount of theanine was determined according to a calibration curve of authentic theanine standard, purchased from Sigma-Aldrich Chemical Company (St. Louis, MO, USA).

### Total RNA Extraction and Real-Time Quantitative RT-PCR Analysis

Total RNA was extracted using a RNAprep Pure Plant Kit (Polysaccharides&Polyphenolics-rich) (TIANGEN, China) according to manufacturer instructions. First-strand cDNA was synthesized using the HiScript^®^ II One Step RT-PCR Kit with gDNA Eraser (Vazyme, China). The 20-µl quantitative real time polymerase chain reaction (qRT-PCR) analysis contained 0.4 µl of forward and reverse primers (10 µM), 1 µl of eight-fold diluted first strand cDNA as template, and 10 µl of SYBR Green Supermix (Vazyme, China). Reactions were performed in a 96-well optical plate at 95°C for 5 min, followed by 40 cycles of 95°C for 10 s and 60°C for 30 s on a Bio-Rad CFX96. A melting curve was generated for each sample after each run to ensure the purity of the amplified products. No-template controls were included for each primer pair in each run. Relative transcript levels were calculated based on the 2^-ΔCt^ method (Schmittgen et al., 2000), using *CsGAPDH* as an internal control. All primers used for qRT-PCR are listed in **Table S4**.

### Identification and Selection of Theanine Biosynthesis and Transport-Related Genes for qRT-PCR Analysis

Theanine biosynthesis pathway genes, including *CsGDHs*, *CsGOGAOTs*, *CsADCs*, *CsTSI*, *CsGGT*, and *CsGSs* were identified and named by Wei et al. (2018). Theanine transport-related genes were identified and named by sequence homology with genes encoding amino acid transporters with glutamine transport activity in *Arabidopsis*. The nucleotide sequences of all these genes were downloaded from a tea plant genome database (Xia et al., 2019, http://teaplant.org/).

### Statistical Analysis

The data were expressed as the mean ± standard deviation (SD) of three independent biological replicates. The correlation coefficient between theanine content and gene expression was analyzed *via* Pearson correlation.

### Yeast Expression, Transformation, and Theanine Transport Measurement

The ORF of *CsLHT* was amplified by PCR from first-strand cDNA of RNA extracted from roots of tea plant (*Camellia sinenesis*, *cv*, Shuchazao) and cloned with HindIII/EcoRI in the yeast expression vector pYES2. The yeast strain 22Δ10α mutant (23344c background) was transformed with pYES2-*CsLHT6* or the empty vector pYES2 using the yeast transformation kit (ZYMO RESEARCH, USA). Transformants were selected on yeast base media (DifcoTM Yeast Nitrogen Base without Amino Acids and Ammonium Sulfate, BD, USA) lacking uracil and supplemented with 2 mM ammonia sulfate.

For theanine transport activity determination, yeast cells were grown on a YNB medium to OD_600_ = 0.8. Yeast cells were collected and washed three times. Before theanine feeding, cells were cultured in YNB liquid medium with no nitrogen source for 1 h, at 30°C. This yeast solution was aliquoted into 4 ml tubes and added to 2 mM or 5 mM theanine for 5 min. The collected yeast cells were washed four times with deionized water. Then, 1 ml deionized water was used to resuspend cells and heated at 98°C for 1 h to extract theanine. After centrifugation at 13,000 rpm for 5 min, the supernatant was collected and filtered through a 0.22 µm membrane for a HPLC-based measurement of theanine.

## Results

### Theanine Accumulation in the Roots of 13 Tea Cultivars During Winter and Spring

To investigate the dynamic theanine accumulation in roots during winter and spring, we measured theanine contents in the roots of 13 tea cultivars on Dec. 12, Mar. 1, Mar. 23, and Apr. 13. The cultivars and the abbreviations of their names are listed in **Figure 1B**. These four time points are representative dates of winter time (Dec.12), leaf bud sprout (Mar. 1), leaf bud harvested for high-quality green tea production (Mar. 23), and leaf quality decreasing for green tea production (Apr. 13), respectively, in the major zone of green tea production in China.

**Figure 2:**
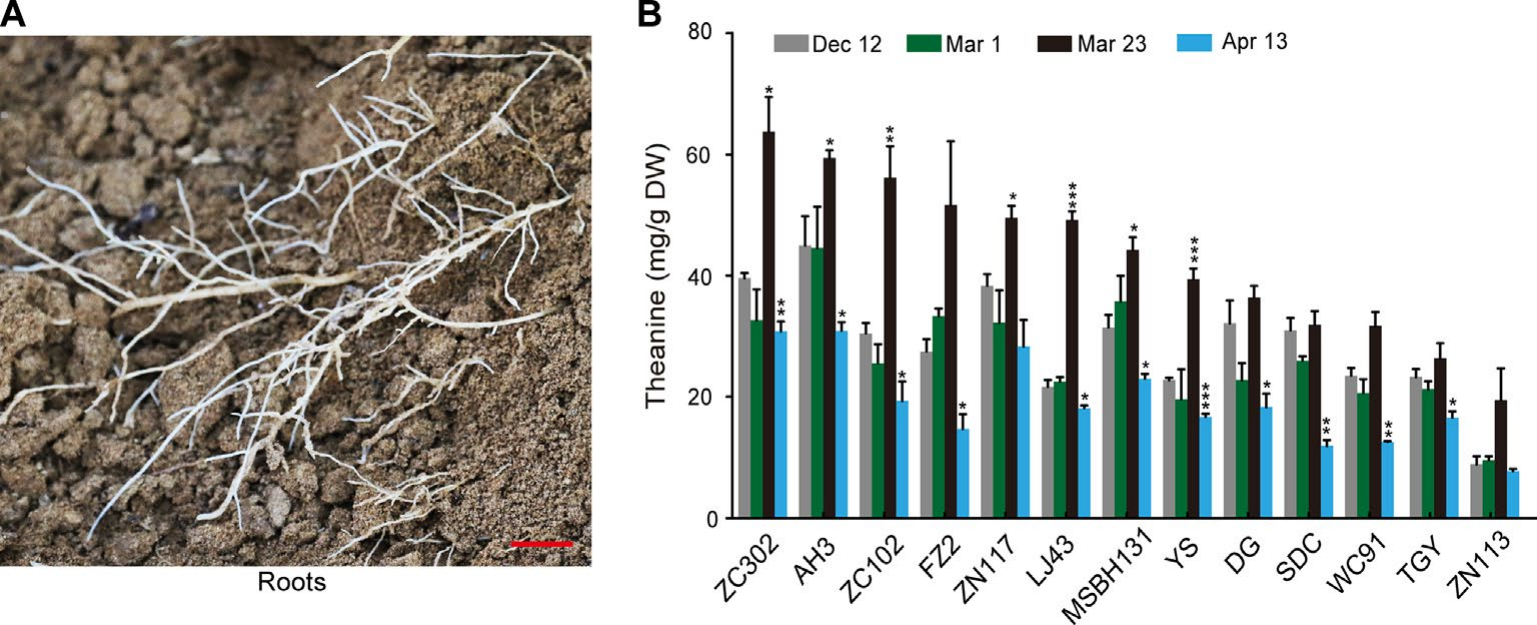
Theanine content in the roots of 13 cultivars at four time points. **(A)** Representative root sample collected. Scale bar: 1 cm **(B)** Theanine content in the roots. Data represent means ± SD of three independent biological replicates. Asterisks represent statistical significance determined by Student’s t-test (*p 0.05, **p 0.01, ***p 0.01).

Interestingly, we observed a consistently changing pattern of theanine content in the roots of these 13 cultivars (**Figures 2A, B**): slightly decreased on Mar. 1 compared with Dec. 12; obviously increased and reached the highest on Mar. 23 in all these cultivars; deceased on Apr. 13. These results suggested theanine accumulation in the roots of the 13 cultivars was dynamically and consistently regulated.

### Expression of Theanine Biosynthesis Pathway Genes in Roots During Winter and Spring

To explore the regulation of the dynamic changes of theanine accumulation in roots, we examined the expression of theanine biosynthesis pathway genes in five cultivars with high (AH3), medium (DG, TGY and Shuchazao [SCZ]), and low (ZN113) levels of theanine in the roots. The contents of theanine in the roots of SCZ at the four time points were reported by Dong et al. (2019). These theanine-biosynthesis genes included three *CsGDHs*, three *CsGOGAOTs*, three *CsADCs*, *CsTSI*, *CsGGT*, and four *CsGSs*, which were named by Wei et al. (2018).

**Figure 3:**
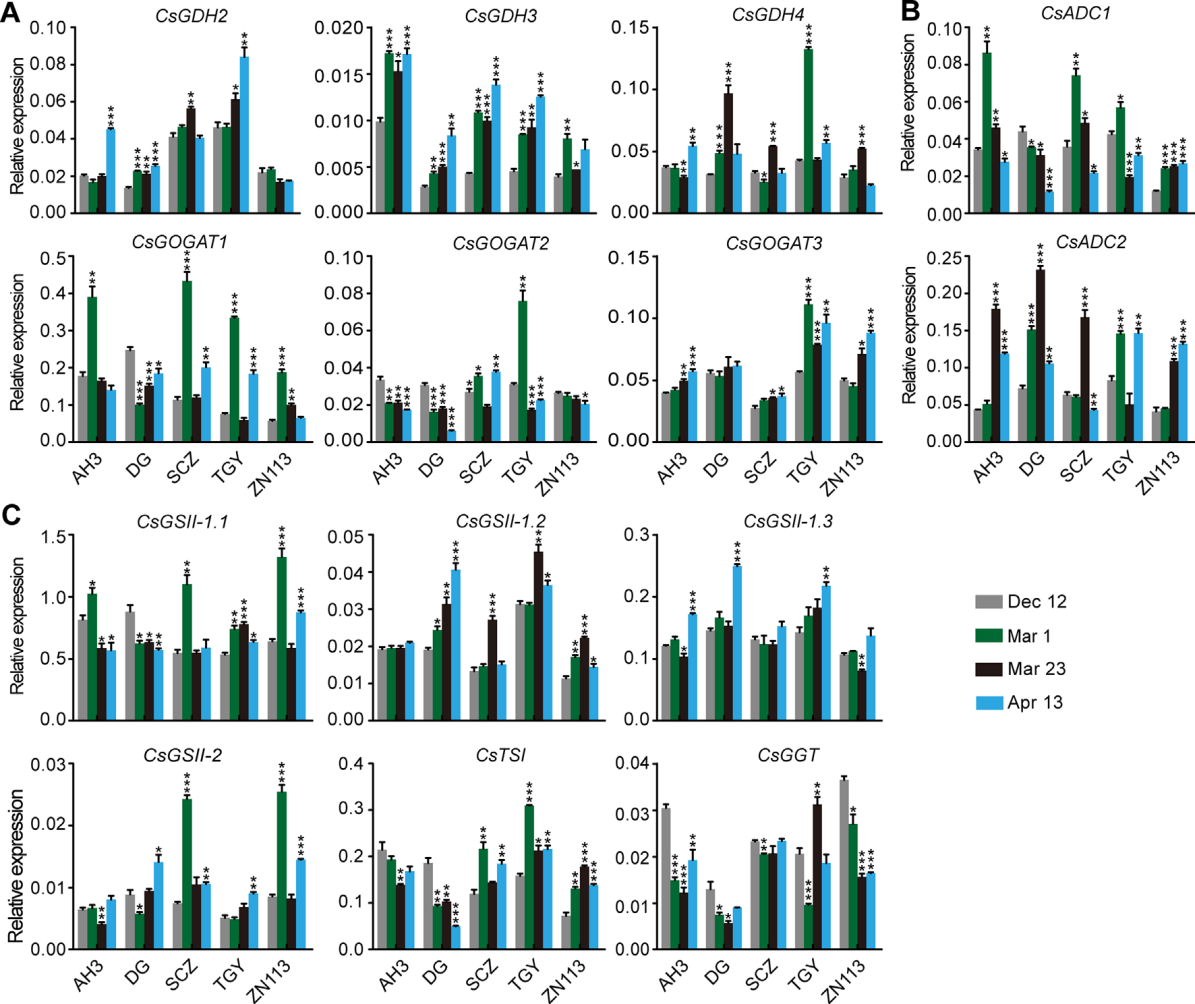
Relative expression of genes in theanine biosynthesis pathway in the roots of five representative cultivars at the four time points. **(A)** Relative expression of *CsGHDs* and *CsGOGATs*. **(B)** Relative expression of genes *CsADCs*. **(C)** Relative expression of *CsGSs*, *CsTSI*, and *CsGGT*. *CsGAPDH* was used as an internal control.Data represent means ± SD of three independent biological replicates. Asterisks represent statistical significance determined by Student’s t-test (*p 0.05, **p 0.01, ***p 0.01).

The expression of genes encoding CsGDHs and CsGOGATs, which catalyze Glu synthesis, was examined first. We found *CsGDHs* were generally increased in spring (Mar. 1, Mar. 23, and Apr. 13) compared with Dec. 12, especially that of *CsGDH2* and *CsGDH3* in these cultivars (**Figure 3A**). Interestingly, *CsGDH4* expression increased on Mar. 1 or Mar. 23 and then reduced on Apr. 13 in most of these cultivars. This changing pattern of *CsGDH4* expression was similar to that of theanine content (**Figure 2B**). By contrast, *CsGOGAT1* expression was highest on Mar. 1 and then reduced on Mar. 23 in four out of five cultivars; next, it increased on Apr. 13 in three out of five cultivars. Thus, the changing pattern of *CsGOGAT1* expression was opposite to that of theanine content during these four time points. In addition, *CsGOGAT2* and *CsGOGAT3* expression was relatively stable during winter and spring. These results indicated that different homologs of *CsGDHs* and *CsGOGATs* respond differently to seasonal factors, suggested that there exist varied roles in glutamate and theanine synthesis during winter and spring in tea plants.

*CsADCs* were proposed to catalyze EA synthesis (Shi et al., 2011). *CsADC1* and *CsADC2* expression was then examined. Interestingly, *CsADC1* expression was generally induced on Mar. 1 and was then gradually repressed on Mar. 23 and Apr. 13, whereas *CsADC2* expression was continuously induced on Mar. 1 and Mar. 23 and then repressed on Apr. 13 in most of these cultivars (**Figure 3B**). Thus, the expression of *CsADC2* generally followed the pattern of theanine content in the roots.

CsTSI and CsGSs are the characterized enzymes catalyzing theanine biosynthesis from Glu and EA (Cheng et al., 2017; Wei et al., 2018). CsGGT is a putative enzyme catalyzing Gln and EA into theanine. Compared with other theanine biosynthesis pathway genes, the expression of *CsGSs*, *CsTS1*, and *CsGGT* was relatively stable in the roots of these cultivars at these four time points (**Figure 3C**), suggesting these genes do not obviously respond to seasonal factors during winter and spring.

### Correlation Between the Expression of Theanine Biosynthesis Pathway Genes and the Theanine Contents in Roots at Each Time Point

**Figure 4:**
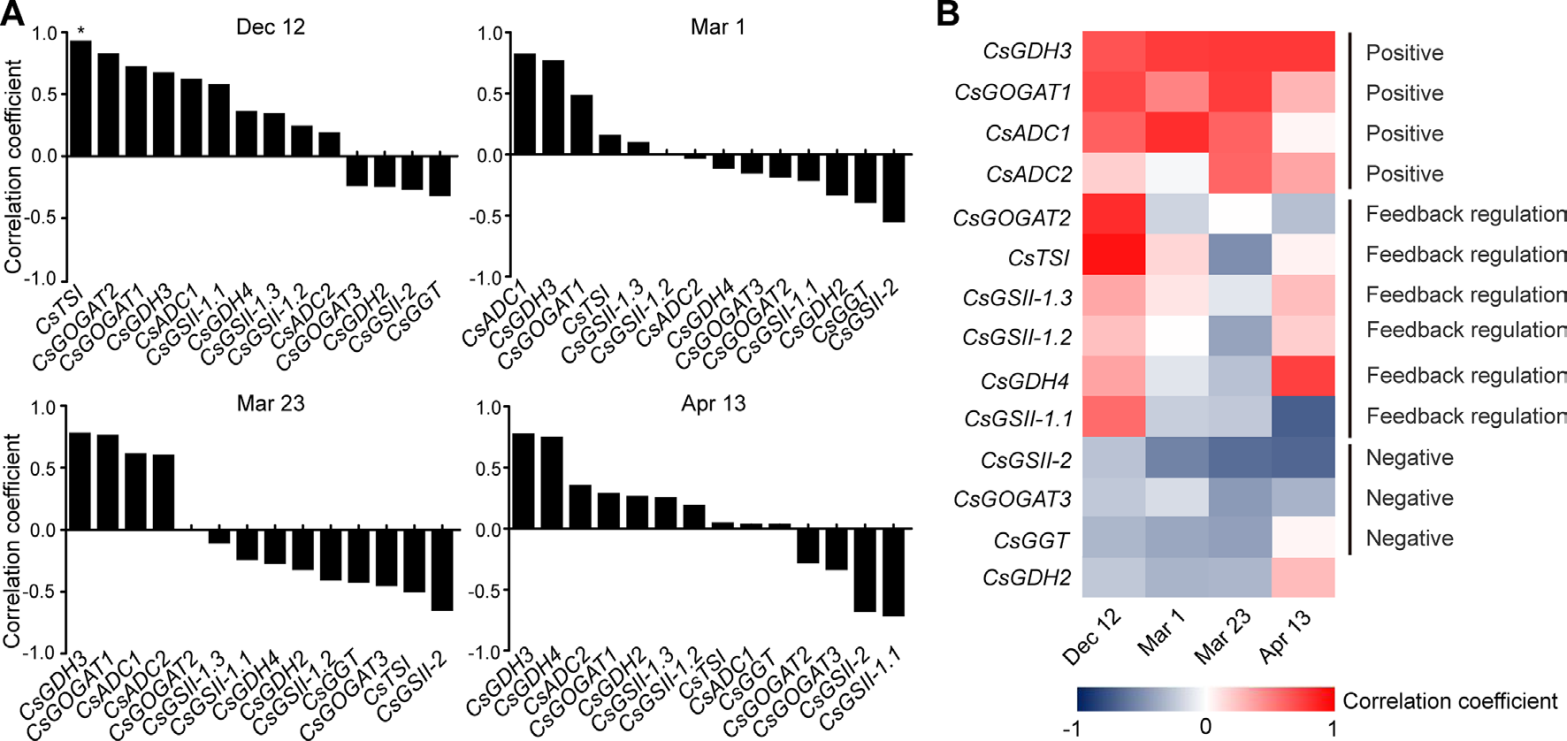
Correlation coefficient between the expression of theanine-biosynthesis pathway genes and the theanine content in the roots of five representative cultivars at each time point. **(A)** Correlation coefficients at each time point. Asterisks represent statistical significance determined by Student’s t-test (*p 0.05). **(B)** Heat map of the Correlation coefficients.

To get further insight into the regulation of theanine biosynthesis in the roots, we next analyzed the correlation between the gene expression and the theanine contents in the roots of AH3, DG, SCZ, TGY, and ZN113 at each time point. The correlation coefficient and *p* values are listed in **Table S1**. Here, we found the expression of these theanine biosynthesis pathway genes was generally and positively correlated with the theanine contents, with 10 out of 14 genes showing positive correlation on Dec. 12 (**Figure 4A**). In great contrast, the expression of most of these genes showed negative correlation with theanine contents on Mar. 1 and Mar. 23. Interestingly, on Apr. 13, most of these genes returned to a positive correlation with theanine contents. Given that the theanine level was relatively lower on Dec. 12, increased in Mar., and decreased on Apr. 13, the pattern of correlation between gene expression and theanine contents suggested theanine biosynthesis is generally feedback regulated in the roots.

Although the expression of the genes examined was generally feedback related with theanine contents in the roots, we found *CsGDH3* and *CsGOGAT1* always positively, and *CsGSII-2* and *CsGOGAT3* always negatively, correlated with theanine content in the roots at all four time points (**Figure 4B**). These results suggested the positive role of *CsGDH3* and *CsGOGAT1* and negative role of *CsGSII-2* and *CsGOGAT3* in theanine synthesis in the roots.

Further, we observed that the expression of *CsGOGAT2* and *CsTSI* showed positive, reduced positive or negative, and higher negative correlation with the theanine contents in the roots on Dec. 12, Mar. 1, and Mar. 23, respectively. Subsequently, *CsTSI* showed a positive correlation with theanine contents on Apr. 13 (**Figure 4B**) when theanine contents decreased in the roots (**Figure 2B**). These results suggested *CsGOGAT2* and *CsTSI* was involved in the feedback regulation of theanine biosynthesis.

### Dynamic Accumulation Pattern of Theanine in the Buds of the 13 Cultivars During Winter and Spring

**Figure 5:**
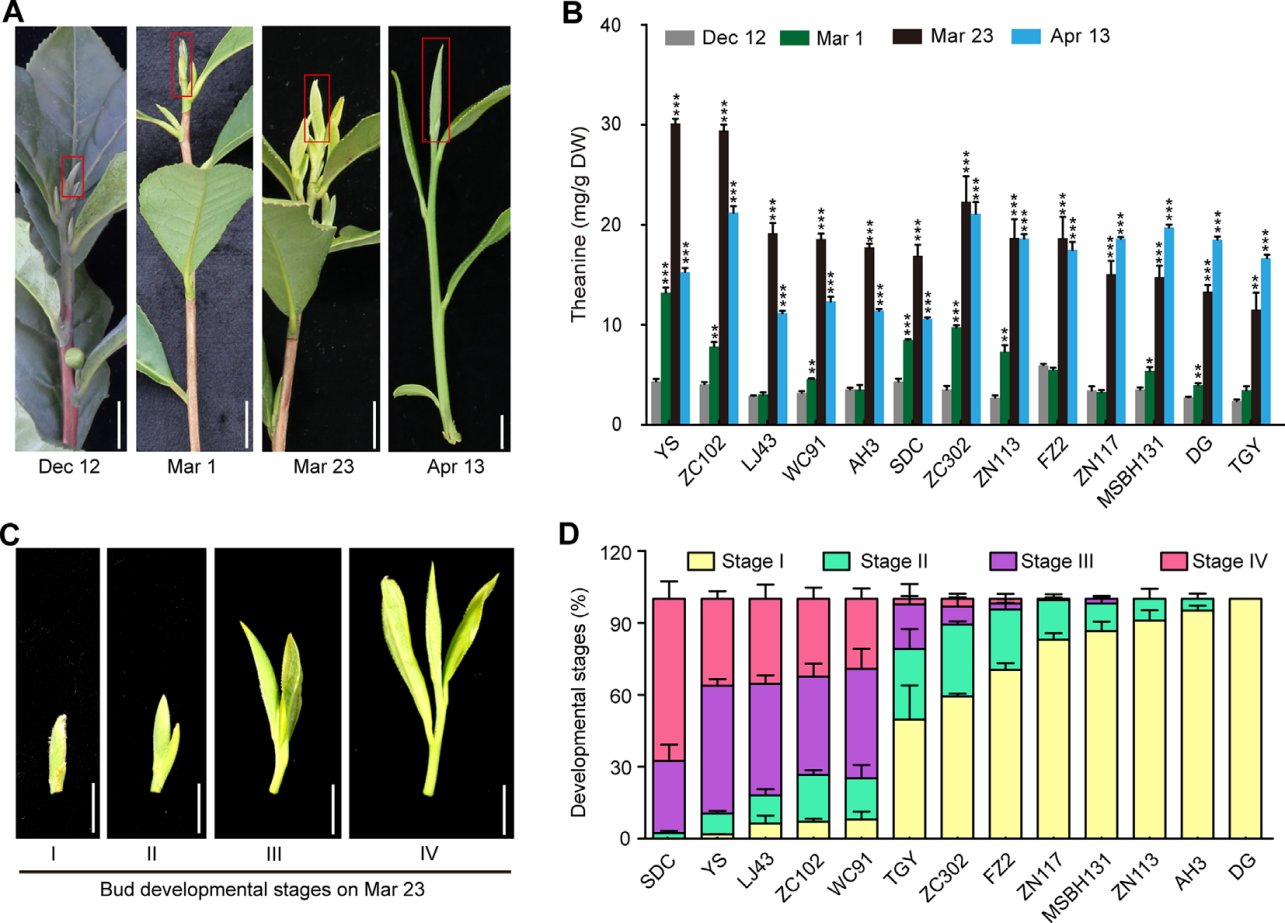
Theanine contents in the buds of 13 cultivars at four time points and the developmental stages of the buds on Mar. 23. **(A)** Representative bud samples collected at four time points. Red boxes highlighted the buds collected. **(B)** Theanine content in the buds at the four time points. Data represent means ± SD of three independent biological replicates. Asterisks represent statistical significance determined by Student’s t-test (*p 0.05, **p 0.01, ***p 0.01). **(C)** Buds of 13 cultivars on Mar. 23 were classified into four developmental stages, as shown. **(D)** Statistic results of the buds of the 13 cultivars at four developmental stages on Mar. 23. Data represent means ± SD of three biological replicates. Scale bars in **(A)** and **(C)**, 1 cm.

Leaf buds contain the highest concentration of theanine within the tissues in shoots (Feldheim et al., 1986) and are the main tissue harvested for high-quality green tea production. To explore how theanine accumulation in leaf buds changes during winter and spring, we next measured the theanine content in the buds of the 13 cultivars at the four time points (Dec. 12, Mar. 1, Mar. 23 and Apr. 13) (**Figure 5A**). We observed that theanine content in the buds was low (∼5 mg/g dry weight) on Dec. 12 in all these cultivars (**Figure 5B**). The content increased on Mar. 1, reached the highest levels on Mar. 23, and decreased on Apr. 13 in most of these cultivars. These results indicated theanine content in the buds generally started to increase in early Mar. and increase to the highest levels in late Mar. or early Apr. before decreasing. This is probably why the quality of green tea produced in Mar. and early Apr. is the highest, only for it to decrease from the middle of Apr.

However, we found that, in ZN117, MSBH131, DG, and TGY, theanine contents in the buds continued to increase on Apr. 13 (**Figure 5B**). In addition, in ZC302, ZN113, and FZ2, theanine content in buds just slightly decreased on Apr. 13 compared with that of Mar. 23. In contrast, in YS, ZN102, LJ43, WC91, AH3, and SDC, theanine content greatly decreased on Apr. 13. We hypothesized that this inconsistency was caused by the different developmental stages of the buds of these cultivars. To test this hypothesis, we evaluated the development stages of buds on Mar. 23 in these cultivars. We defined bud developmental stages I, II, III, and IV, as illustrated in **Figure 5C**. Interestingly, we found most of the buds of YS, ZN102, LJ43, and WC91 were in Stage III and IV, suggesting an early sprout time for the buds of these cultivars. However, most of the buds of ZN117, MSBH131, DG, TGY, ZC302, ZN113, and FZ2 were in Stage I and II (**Figure 5D**), suggesting a late sprout time for the buds of these cultivars. Therefore, the dynamic change of theanine content in these buds is highly associated with both seasons and bud developmental stages.

### Expression of Theanine Biosynthesis Pathway Genes in Buds During Winter and Spring

Although theanine is mainly synthesized in the root, Deng and colleagues showed that tea plant shoots also have a weak theanine synthesis capacity (Deng et al., 2009). To explore the molecular mechanism of the dynamic changes of theanine in the buds, we next investigated the expression of theanine biosynthesis pathway genes in the buds of AH3, ZN113, TGY, DG, and SCZ. Theanine content in the buds of SCZ at the four time points was reported by Dong et al. (2019).

**Figure 6:**
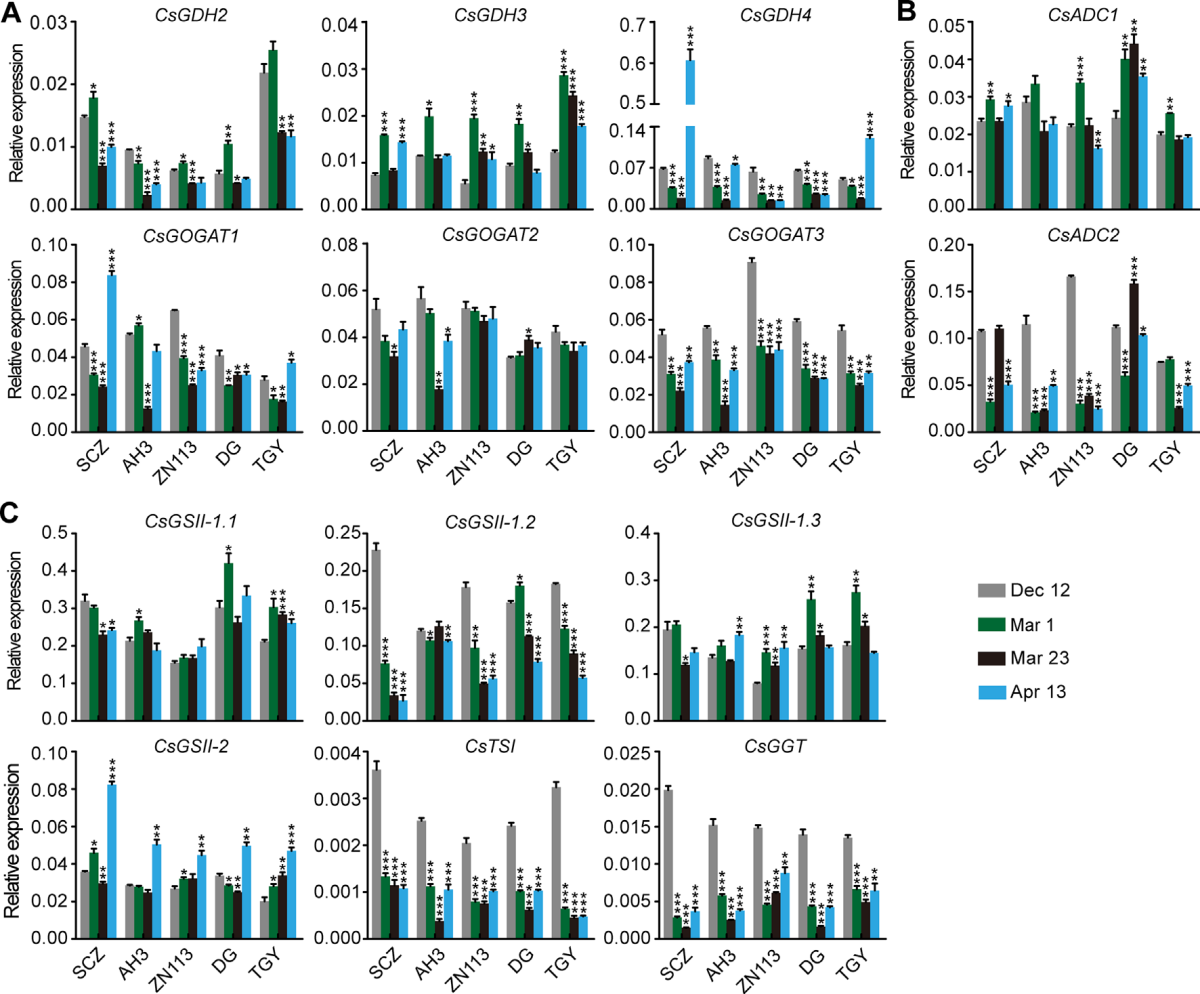
Relative expression of genes in theanine-biosynthesis pathway in the buds of five representative cultivars, at the fourtime points. **(A)** Relative expression of *CsGHDs* and *CsGOGATs*. **(B)** Relative expression of genes *CsADCs*. **(C)** Relative expression of *CsGSs*, *CsTSI*, and *CsGGT*. *CsGAPDH* was used as an internal control. Data represent means ± SD of three independent biological replicates. Asterisks represent statistical significance determined by Student’s t-test (*p 0.05, **p 0.01, ***p 0.01).

We observed that the expression of *CsGDH4*, *CsGOGAT1*, *CsGOGAT3*, *CsTSI*, and *CsGGT* was suppressed on Mar. 1 and Mar. 23 and was increased on Apr. 13 (**Figures 6A–C**). These results—indicated in the changed pattern of the expression of these genes—were generally opposite to those of the theanine content in the buds (**Figure 5B**) and implied that the expression of these genes was probably feedback regulated by theanine content in the buds.

In contrast to the feedback-regulated genes, *CsGSII-1.2* was gradually repressed in the buds from Dec. 12 to Apr. 13 (**Figure 6C**). *CsGSII-2* expression, however, was stable on Dec. 12, Mar. 1, and Mar. 23 and was induced on Apr. 13 in all five cultivars. Therefore, *CsGSII-1.2* and *CsGSII-2* expression in the buds was likely regulated by seasonal factors, including temperature and light, but was not regulated by the theanine content in buds. In addition, *CsGOGAT2* and *CsGSII-1.1* expression in the buds was generally stable at all these four time points (**Figures 6A, C**). This result suggested *CsGOGAT2* and *CsGSII-1.1* was likely not regulated by theanine content in the bud.

### Correlation Between the Expression of Theanine Biosynthesis Pathway Genes and Theanine Content in the Buds at Each Time Point

**Figure 7:**
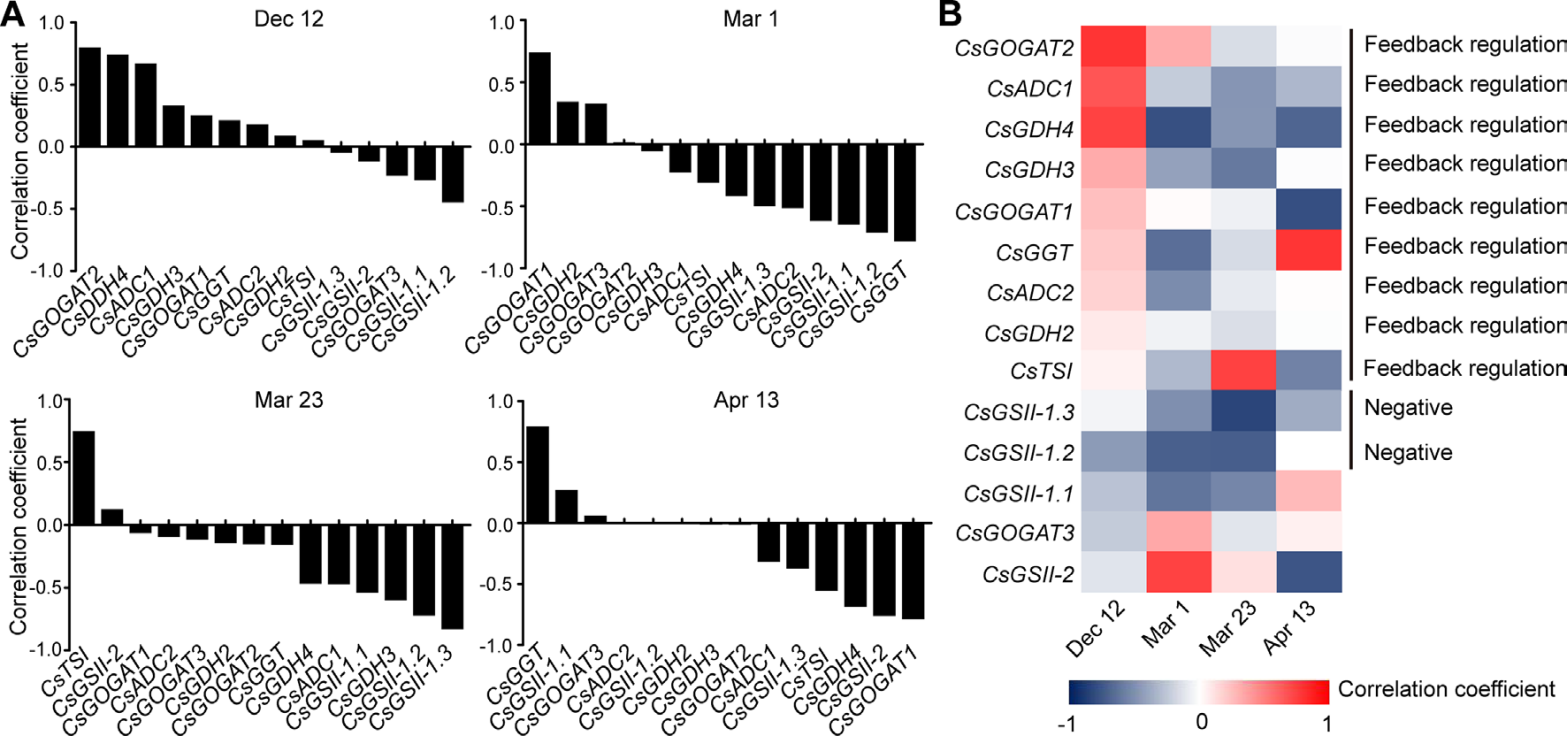
Correlation coefficient between the expression of theanine-biosynthesis pathway genes and the theanine content in the buds of five representative cultivars at each time point. **(A)** Correlation coefficients at each time point. Statistical analysis was performed and there was no statistical significance, but the correlation pattern is consistent and valuable. **(B)** Heat map of the correlation coefficients.

To investigate the regulation of theanine-biosynthesis gene expression in the buds, we also analyzed the correlation between gene expression and theanine content at each time point. The correlation coefficient and *p* values are listed in **Table S2**. Similarly to the situation in the roots, most of the genes had a positive correlation with theanine content in the buds (**Figure 7A**), when theanine content in the buds was low; next, 10 and 12 out of 14 genes had a negative correlation on Mar. 1 and Mar. 23, when theanine content in the buds increased, respectively. Furthermore, the number of genes that had a negative correlation reduced to six on Apr. 13 when theanine content decreased. These results suggested the expression of theanine biosynthesis pathway genes was also generally feedback regulated by the theanine content in buds.

Next, we analyzed the correlation of individual genes. We observed that *CsGDH2/3/4*, *CsGOGAT1/2*, *CsADC1/2*, and *CsGGT* showed positive correlation when theanine content was low, negative correlation when theanine content increased, and positive correlation when theanine content decreased in the buds (**Figure 7B**). These results suggested that the expression of these eight genes was typically feedback regulated by theanine levels in the buds.

Interestingly, *CsGSII-1.1/2/3* was almost always negatively correlated with theanine contents in the buds (**Figure 7B**), suggesting a negative role of these three genes in theanine biosynthesis in the buds. Finally, the fact that no gene showed continuously positive correlation with theanine contents in the buds supported the notion that buds have a minor role in theanine biosynthesis in tea plants.

### Expression of Amino Acid Transporter Genes in the Roots During Winter and Spring

Amino acid long-distance transport is mediated by amino acid transporters (Tegeder, 2014). However, the molecular mechanism of theanine root-to-shoot transport is still largely unknown. To explore this mechanism, we next examined the expression of amino acid transporter genes in the roots of the five cultivars. These examined amino acid transporter genes included members of AAP, LHT, ProT, PUT, AUX, UMAMIT, VAAT, and ATL families.

**Figure 8:**
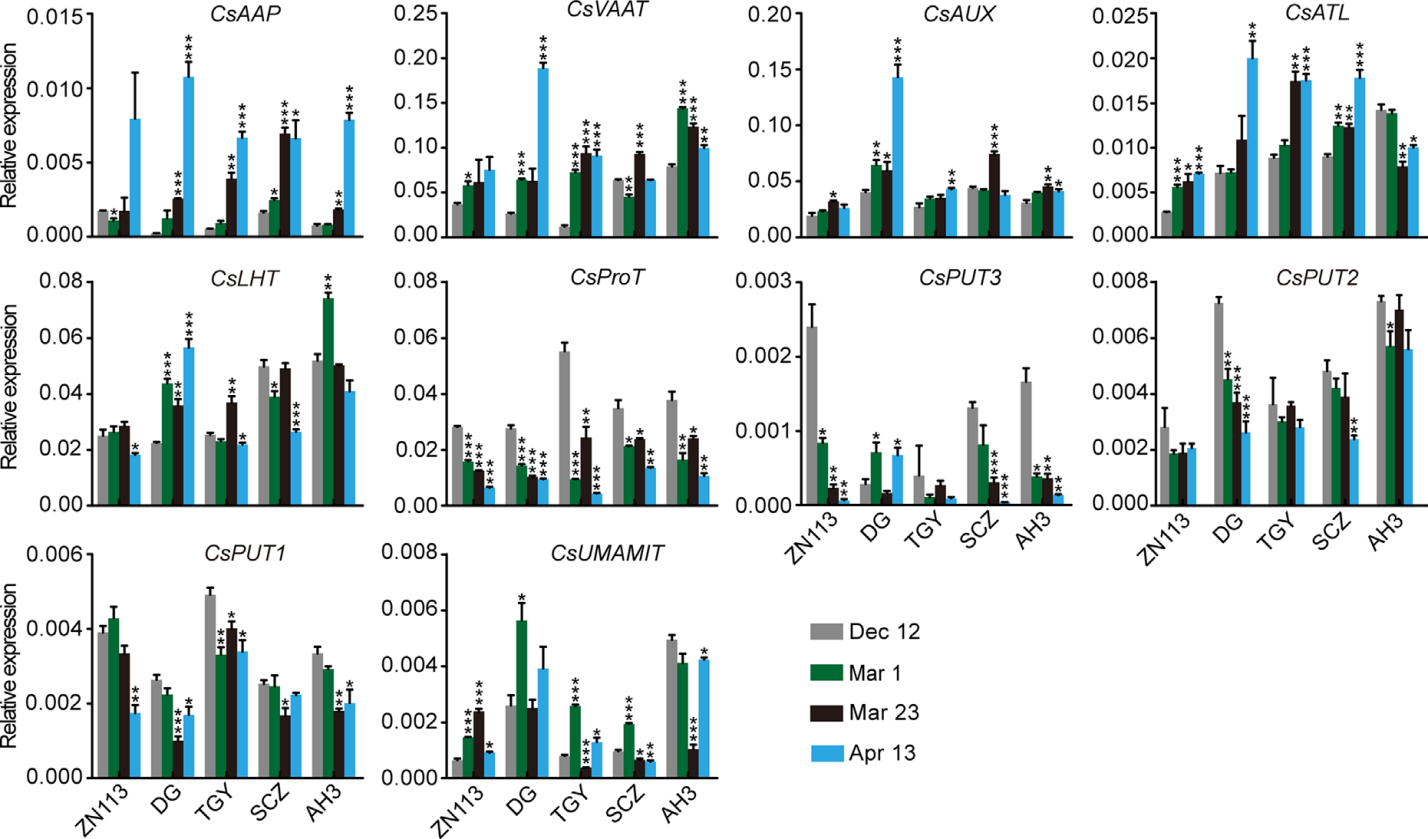
Relative expression of amino acid transporter genes in the roots of five representative cultivars, at the four time points. *CsGAPDH* was used as an internal control. Data represent means ± SD of three independent biological replicates. Asterisks represent statistical significance determined by Student’s t-test (*p 0.05, **p 0.01, ***p 0.01).

We found that the expression of *CsAAP9*, *CsVAAT*, *CsAUX*, and *CsATL* was gradually induced in the roots of most of these cultivars through the four time points (**Figure 8**). By contrast, the expression of *CsProT* and *CsPUT2* was gradually repressed. Although *CsLHT* was expressed at different levels in these cultivars, its expression levels were relatively stable through the four time points. Similarly, *CsAAP2* expressed differentially but stably in these cultivars on Dec. 12, Mar. 1, and Mar. 23, but was great repressed on Apr. 13.

It is worth noting that theanine content in roots as slightly decreased on Mar. 1, increased on Mar. 23, and then decreased on Apr. 13 (**Figure 2B**). Thus, the change in the expression of these genes was generally different from that of theanine content in the roots. One exception was *CsUMAMIT*; this was generally induced when theanine content decreased and was repressed when theanine content increased in the roots (**Figure 8**), suggesting feedback regulation of its expression by theanine content in the root.

### Correlation of Amino Acid Transporter Gene Expression With Theanine Content in the Roots During Winter and Spring

**Figure 9:**
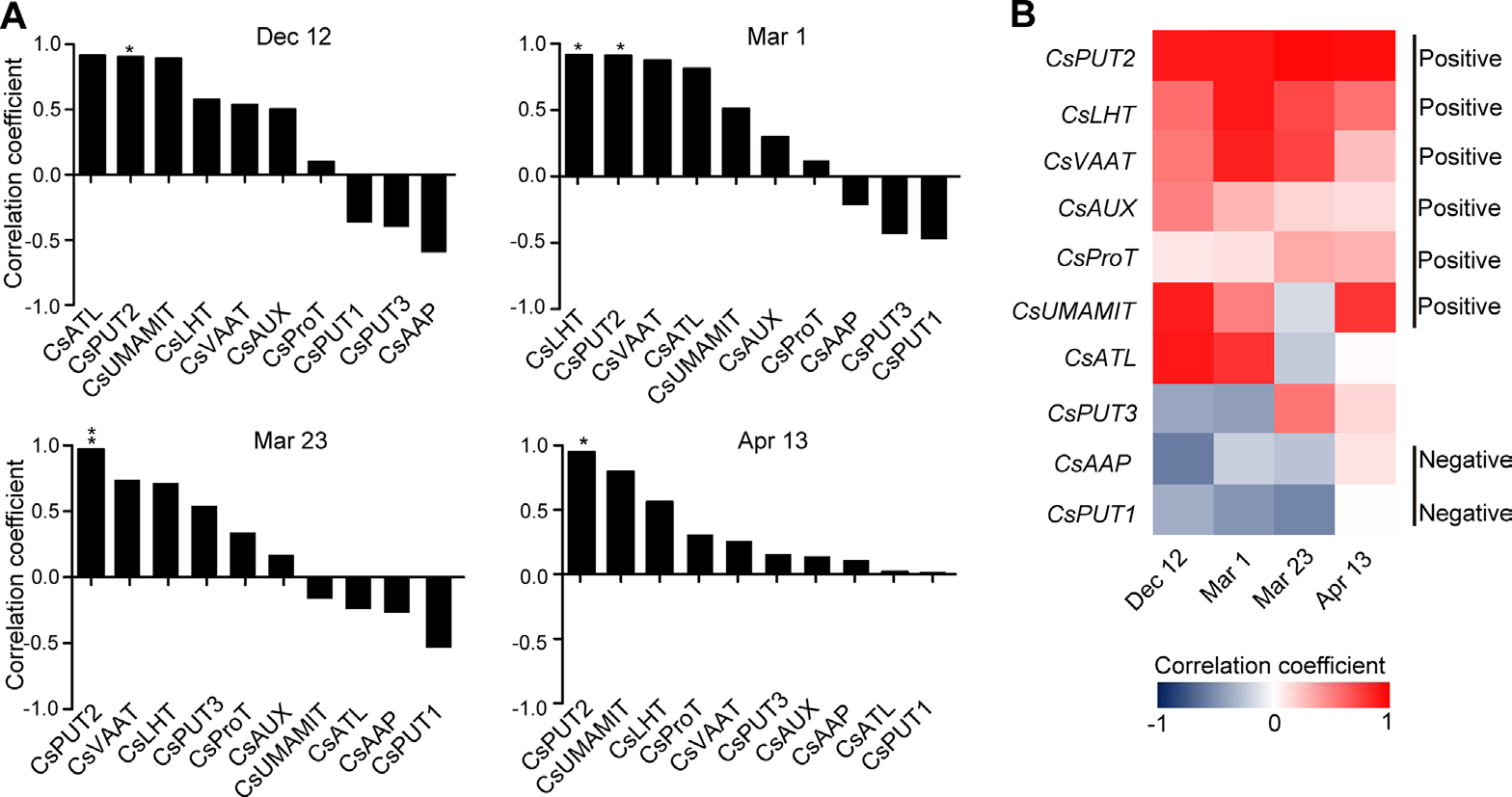
Correlation coefficient between the expression of amino acid transporter genes and the theanine contents in the roots of five representative cultivars at each time point. **(A)** Correlation coefficients at each time points. Asterisks represent statistical significance determined by Student’s t-test (*p 0.05, **p 0.01). **(B)** Heat map of the correlation coefficients.

To further investigate the regulation of theanine transport, we next analyzed the correlation of *CsAATs* expression with theanine content at each time point in the roots of the five representative cultivars. The correlation coefficient and *p* values are listed in **Table S3**. Different from theanine biosynthesis pathway genes, most of the *CsAATs* showed positive correlation with theanine content in the roots at all these four time points (**Figures 9A, B**).

When analyzed individually, we found *CsPUT2* was always highly and positively correlated with theanine content in the roots at these four time points (**Figures 9A, B**), suggesting a continuous positive role of CsPUT2 in theanine transport. Although *CsLHT* and *CsVAAT* had positive correlation at all the four time points, they had higher positive correlation in Mar. and lower positive correlation on Apr. 13. This result suggested the positive role of CsLHT and CsVAAT in theanine transport was feedback promoted. Although most of these genes positively correlated with theanine contents in the roots, *CsPUT1*and *CsAAP9* had negative correlation on Dec. 12, Mar. 1, and Mar. 23, suggesting CsPUT1 and CsAAP had a negative role in theanine transport.

### CsLHT Can Transport Theanine

Theanine content in xylem sap increases in spring (Ruan et al., 2012), suggesting there is root-derived theanine transport to the shoot *via* the xylem. To explore the molecular mechanism of theanine root-to-shoot transport, it is necessary to identify what theanine transporter mediated this process. Given that *CsPUT*, *CsLHT*, and *CsVAAT* expression was positively correlated with theanine content in the roots (**Figure 9B**), CsPUT, CsLHT, and CsVAAT were candidates of theanine transporters. As LHT-family amino acid transporters have a high affinity for neutral and acidic amino acids and have been well studied in plants (Tegeder and Ward, 2012), we then examined whether CsLHT can transport theanine.

**Figure 10:**
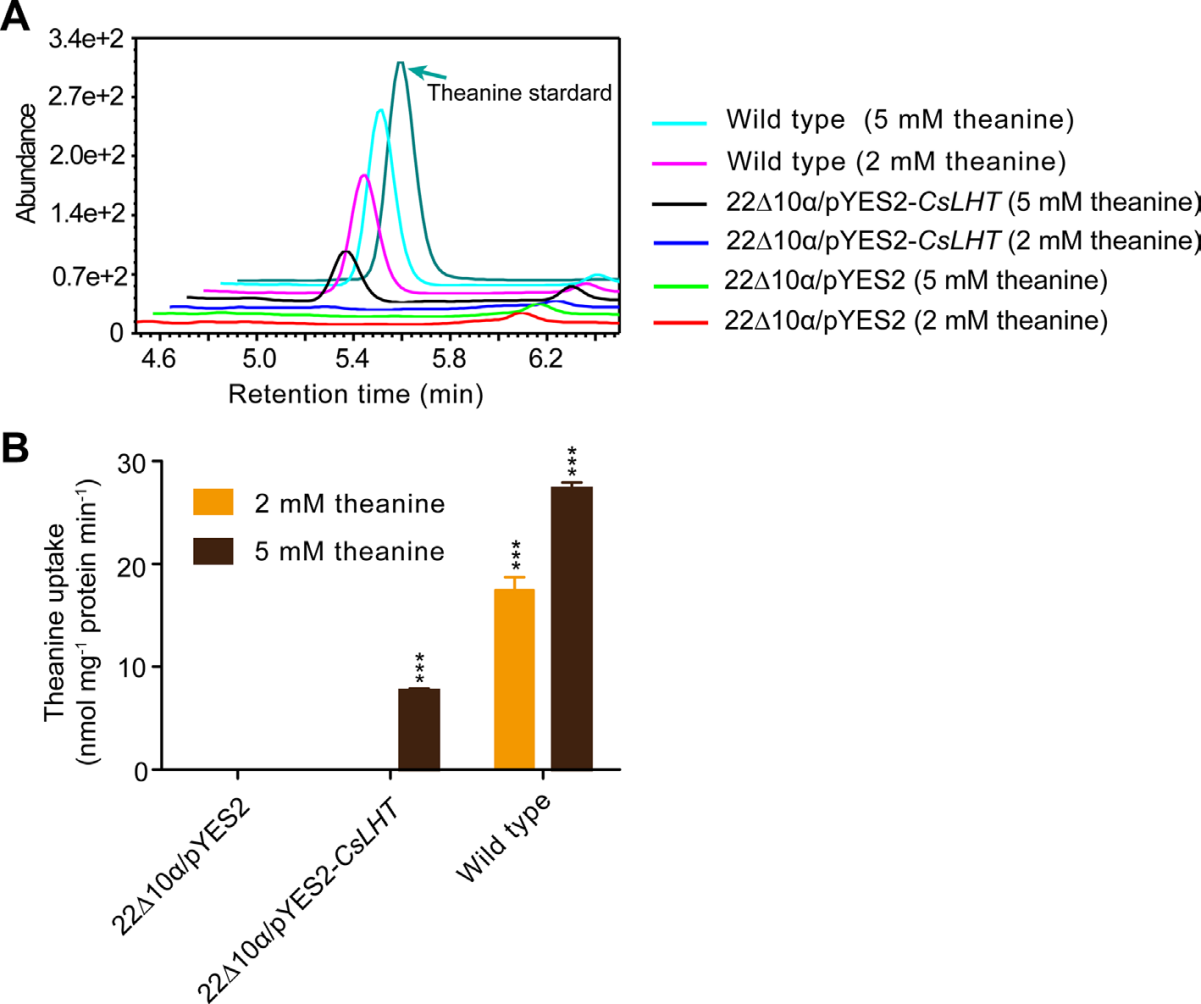
CsLHT transported theanine into yeast cells. **(A)** HPLC identification theanine in the yeast mutant strain 22Δ10α mediated by CsLHT. Yeast transformed with empty vector was used as a negative control and wild type yeast strain (23344c) as a positive control. **(B)** Determination of the theanine contents in 22Δ10α/pYES2, 22Δ10α/pYES2-*CsLHT* and wild-type yeast cells. Data represent means ± SD of three independent biological replicates. Asterisks represent statistical significance determined by Student’s t-test (***p 0.01).

The yeast 22∆10∆α strain is an amino acid uptake mutant lacking an amino acid uptaking capacity due to the mutations of 10 amino acid transporter genes (Besnard et al., 2016). To test the theanine transport activity of CsLHT, we expressed *CsLHT* in 22∆10∆α with an empty vector pYES2 as a negative control and wild-type yeast strain 223344c as a positive control. Under the feeding of 2 or 5 mM theanine, we measured contents of theanine accumulated in these yeast strains. We found wild-type yeast accumulated the highest levels of theanine under both 2 and 5 mM theanine feeding (**Figures 10A, B**); *CsLHT*-expressing yeast cells only accumulated theanine under 5 mM theanine feeding. No theanine accumulation, however, was detected in the negative control strain. These results indicated that CsLHT can transport theanine under a higher theanine concentration.

## Discussion

### Theanine Accumulation Pattern in Root and Bud of Tea Plant in Winter and Spring

As one of the most important components of quality in green tea, theanine and its biosynthesis has been studied intently in tea plants since it was discovered by Yajiro Sakato in 1949 (Ashihara, 2015; Mu et al., 2015). These studies indicated that theanine accumulation was dynamically regulated by seasons (Konishi and Takahashi, 1969; Takeo, 1981; Janet et al., 2015), light (Yang et al., 2012; Deng et al., 2013; Chen et al., 2017; Ji et al., 2018; Sano et al., 2018), nutrient levels (Takeo, 1981; Ruan et al., 2007; Ruan et al., 2010; Ruan et al., 2012; Huang et al., 2018; Kc et al., 2018; Zhu et al., 2019), hormones (Li et al., 2016a), developmental stages (Feldheim et al., 1986; Li et al., 2016b; Liu et al., 2017), and stress (Deng et al., 2012; Wang et al., 2016). Theanine accumulation is also highly tissue-and cultivar-dependent (Fang et al., 2017; Liu et al., 2017; Chen et al., 2018a; Chen et al., 2018b). Theanine accumulation, therefore, is dynamic and highly regulated.

Most of the studies on theanine accumulation under various conditions were performed in one cultivar. In addition, when examining the effects of seasons, comparisons were made between spring, summer, and autumn. Furthermore, because new shoots or tender leaves are the harvested targets for tea, most of the studies focused on theanine accumulation in leaves. However, how theanine accumulation in roots and new shoots changes, during winter and spring, in various cultivars remains to be observed.

In this study, we clearly observed that theanine content in the roots of 13 cultivars slightly deceased or kept stable from Dec. 12 to Mar. 1 and then increased from Mar. 1 to Mar. 23, followed by an obvious decrease on Apr. 13 (**Figure 2**). This is slightly different from the Takeo’s report in 1979. He observed that the theanine content in roots slightly increased from Jan. to Feb. and then continuously decreased from Feb. to May; he subsequently concluded that theanine was mainly accumulated in winter. Our results, however, suggested that theanine content in roots is highest in late of Mar. or early Apr. and then decreases from the middle of Apr. This inconsistency may be caused by geographical factors or the origin area of the cultivars (Lee et al., 2010; Chen et al., 2018a). In addition, our results also indicated that the theanine levels in roots are likely controlled by genetic background; however, the changing pattern is mainly regulated by seasonal factors (**Figure 2**).

Different from that in roots, theanine content in buds was low on Dec. 12 and started to increase on Mar. 1 (**Figure 5**). Theanine content reached the highest in most of the cultivars on Mar. 23 and decreased on Apr. 13. But, in some cultivars, theanine content kept increasing on Apr. 13. Further analyses indicated the different changing patterns of theanine content on Mar. 23 and Apr. 13 were associated with bud sprout time and developmental stages. Therefore, theanine content in buds is regulated by genetic background, seasonal factors, and developmental stages. Here, it is also noteworthy that theanine content decreased quickly in April, and this is highly associated with the decline in green tea quality made from the new buds.

### Regulation of the Expression of Theanine Biosynthesis and Amino Acid Transporter Genes

Many previous studies showed that the expression of theanine biosynthesis pathway genes was not always positively correlated with theanine content (Ruan et al., 2010; Fang et al., 2017; Kc et al., 2018; Zhu et al., 2019). Kc et al. found the expression of most theanine biosynthesis pathway genes was negatively correlated with theanine content in leaves (Kc et al., 2018). These results led us to explore the regulation of the expression of theanine biosynthesis and transport pathway genes in response to dynamic theanine accumulation in roots and buds.

In this study, we analyzed the correlation between the expression of theanine biosynthesis pathway genes and theanine content in roots and buds at four time points. These results demonstrated that the expression of theanine biosynthesis pathway genes is generally feedback correlated with theanine content in both roots and buds of tea plants (**Figures 4** and **7**). These results suggested that the expression of theanine biosynthesis genes is generally feedback regulated at transcription level by theanine levels. This is consistent with the regulation of amino acid biosynthesis genes in model plants (Pratelli and Pilot, 2014).

Although the expression of genes in theanine-synthesis pathways is generally feedback regulated in both roots and buds, the feedback regulation in buds is more profound than in roots. In the 14 genes examined, eight genes were feedback regulated in buds; in contrast, the number was six in the roots (**Figures 4** and **7**). More importantly, four genes were always positively correlated with theanine contents in the roots; however, no gene that was always positively correlated was identified in buds.

The molecular mechanisms of theanine transport are largely unknown in tea plants. In this study, we found the expression of most amino acid transporter genes examined was positively correlated with theanine content in the roots. These results implied that amino acid transporter genes are induced by theanine accumulation. This result also provided candidate genes for identifying theanine transporters.

### Putative Key Genes Involved in the Regulation of the Dynamic Biosynthesis

In this study, we observed that the expression of genes in the same family correlated differently with theanine contents (**Figures 4**, **7**, **9**). For example, *CsGDH3* showed continuously positive correlation with theanine content in the roots; *CsGDH4*, however, was always negatively correlated with theanine content in the roots. Similar correlation results were also observed by other reports (Li et al., 2016b; Liu et al., 2017; Zhu et al., 2019). These results indicated that genes in the same family were differently regulated and also suggested varied roles for these genes in theanine biosynthesis and transport.

In addition to this, the correlation degrees were also varied for different genes in theanine biosynthesis pathways and amino acid transporters (**Figures 4**, **7** and **9**). These results suggested *CsGDH3*, *CsGOGAT1*, *CsADC1*, and *CsADC2* have important roles in the positive regulation of theanine biosynthesis in roots by providing glutamate and ethylamine, two direct substrates of theanine biosynthesis.

### CsLHT May Mediate Theanine Transport in Tea Plants

The high correlation of *CsPUT2*, *CsLHT*, and *CsVAAT* with theanine content in roots suggested these amino acid transporters have important roles in theanine transport. We further showed that CsLHT can transport theanine in yeast under 5 mM theanine feeding. This result supports the argument that CsLHT is a low affinity transporter for theanine. Considering that theanine content in roots is very high (∼60 mg/g dry weight, ∼150 mM/kg fresh weight), it is reasonable to have low affinity transport to mediate theanine transport. We will further biochemically characterize CsLHT in terms of its affinity for theanine and its function in mediating theanine root-to-shoot transport in the future. It is also interesting to test the theanine transport activity of CsPUT and CsVAAT.

Taken together study first uncovered the dynamic accumulation pattern of theanine in the roots and buds of tea plants during winter and spring. Next, the expression of theanine biosynthesis and transport genes in response to dynamic theanine accumulation was investigated in the roots and buds of 13 tea plant cultivars at four time points in winter and spring. Finally, a theanine transporter was identified in tea plants. These findings provided fundamental bases for further revealing the regulation mechanisms of theanine biosynthesis and transport in tea plants.

## Data Availability Statement

All datasets generated for this study are included in the article/**Supplementary Material**.

## Author Contributions

ZZ, XW, and CW conceived the study and designed the experiments. FL, CD, TY, JM, and SZ carried out the experiments. FL, CD, TY, and ZZ analyzed the data. ZZ, XW, and FL wrote the paper. All authors read and approved the final manuscript.

## Funding

This work was supported by the National Key R&D Program of China (2018YFD1000601) and grants from the Department of Science and Technology of Anhui Province (17030701049 to ZZ) and the National Natural Science Foundation of China (31770731 to ZZ).

## Conflict of Interest

The authors declare that the research was conducted in the absence of any commercial or financial relationships that could be construed as a potential conflict of interest.
